# Rapid Generation of MicroRNA Sponges for MicroRNA Inhibition

**DOI:** 10.1371/journal.pone.0029275

**Published:** 2012-01-06

**Authors:** Joost Kluiver, Johan H. Gibcus, Chris Hettinga, Annelies Adema, Mareike K. S. Richter, Nancy Halsema, Izabella Slezak-Prochazka, Ye Ding, Bart-Jan Kroesen, Anke van den Berg

**Affiliations:** 1 Department of Pathology and Medical Biology, University Medical Center Groningen, Groningen, The Netherlands; 2 Wadsworth Center, New York state Department of Health, Albany, New York, United States of America; National Institute of Health, United States of America

## Abstract

MicroRNA (miRNA) sponges are transcripts with repeated miRNA antisense sequences that can sequester miRNAs from endogenous targets. MiRNA sponges are valuable tools for miRNA loss-of-function studies both *in vitro* and *in vivo*. We developed a fast and flexible method to generate miRNA sponges and tested their efficiency in various assays. Using a single directional ligation reaction we generated sponges with 10 or more miRNA binding sites. Luciferase and AGO2-immuno precipitation (IP) assays confirmed effective binding of the miRNAs to the sponges. Using a GFP competition assay we showed that miR-19 sponges with central mismatches in the miRNA binding sites are efficient miRNA inhibitors while sponges with perfect antisense binding sites are not. Quantification of miRNA sponge levels suggests that this is at least in part due to degradation of the perfect antisense sponge transcripts. Finally, we provide evidence that combined inhibition of miRNAs of the miR-17∼92 cluster results in a more effective growth inhibition as compared to inhibition of individual miRNAs. In conclusion, we describe and validate a method to rapidly generate miRNA sponges for miRNA loss-of-function studies.

## Introduction

MicroRNAs (miRNAs) are involved in a multitude of biological processes and diseases. For an in-depth understanding of miRNA function it is imperative to have proper tools for gain- or loss-of-function studies. Several methods have been developed to increase miRNA expression, including miRNA precursor transfection, (viral) vector based overexpression and the generation of transgenic animals. For miRNA loss-of-function, the use of miRNA antisense inhibitor oligonucleotides, knockouts and miRNA sponges have been described [1]–[5]. Each of the techniques for inhibition of miRNA activity has its specific advantages and disadvantages. Inhibitor oligonucleotides are especially useful for short term experiments while gene-knockouts allow studying the role of miRNAs *in vivo*. However, the generation of knockout mice is time consuming, costly and technically challenging as a large percentage of miRNAs are located within protein-coding genes or are part of a miRNA cluster. MiRNA sponges, i.e. *in vivo* expressed transcripts that contain multiple miRNA antisense binding sites (MBS) to sequester miRNAs, have been described as an alternative to the generation of knockouts [6]–[8]. In the first paper on miRNA sponges by Ebert and colleagues it was shown that introduction of miRNA sponges resulted in de-repression of miRNA targets *in vitro* [5]. In *Drosophila melanogaster* miRNA sponges could accurately copy the phenotype of loss-of-function mutants although the effects were milder [9]. A sponge for miR-223 in bone marrow transplantation assays could phenocopy several characteristics of a miR-223 knockout mouse [10].

Several miRNAs have seed family members at different loci that may have overlapping functions. Studying the role of such miRNAs using loss-of-function mutants may be difficult as all of these loci may need to be targeted to accurately study its function. MiRNA sponges can potentially inhibit all seed family members of a miRNA and thus offers the additional advantage of studying the function of a miRNA seed family. Furthermore, by introducing multiple different MBS, e.g. MBS for all miRNAs of a specific miRNA cluster, sponge technology can also be used to study the role of different miRNAs simultaneously.

Sponges with an imperfect MBS, i.e. a MBS that include a 4 nucleotide (nt) central bulge (“bulged sponges”), are reported to be more effective for the sequestration of miRNAs than sponges with perfect antisense MBS [5], [10], [11]. This may be caused by degradation of the sponge transcripts due to endonucleolytic cleavage activity of AGO2 upon perfect binding of the miRNA [12], [13]. On the other hand, several other studies have reported efficient inhibitory activity of perfect antisense sponges [5], [10], [14], [15]. The number of MBS in a sponge is also crucial for their effectiveness [16], [17]. More MBS increases the likelihood of reaching maximal miRNA sequestration but it may also increase the chance of sponge transcript degradation.

Two different strategies have been described for cloning of miRNA sponges containing multiple MBS. The first approach is based on the non-directional concatemerization of oligo duplexes followed by the subsequent ligation of 5′ and 3′adapters [5]. The resulting products are gel-purified, digested with the appropriate restriction enzymes and cloned to the vector. In the second approach long oligos that allow 2 (∼50-mers) or 4 MBS (∼100-mers) are designed with appropriate overhangs to allow direct directional cloning [7], [16]. Although functional sponges can be generated with these methods they both entail drawbacks. The first method is relatively labor intensive and inefficient due to the non-directional cloning approach. The second method allows incorporation of only a limited number of MBS in the miRNA sponge due to size limitations and is relatively expensive due to the extraordinary length of such oligos.

Here, we describe and validate a protocol that allows rapid and efficient generation of miRNA sponges with varying sizes using a single ligation reaction. We tested the effectiveness of these bulged and perfect sponges with different numbers of MBS in reporter and proliferation assays. In addition, we also used a minigene approach to inhibit all individual members of the miR-17∼92 cluster simultaneous and show that combined inhibition of all miRNAs of this cluster results in a more severe phenotype than inhibition of individual miRNAs.

## Results

**Figure 1:**
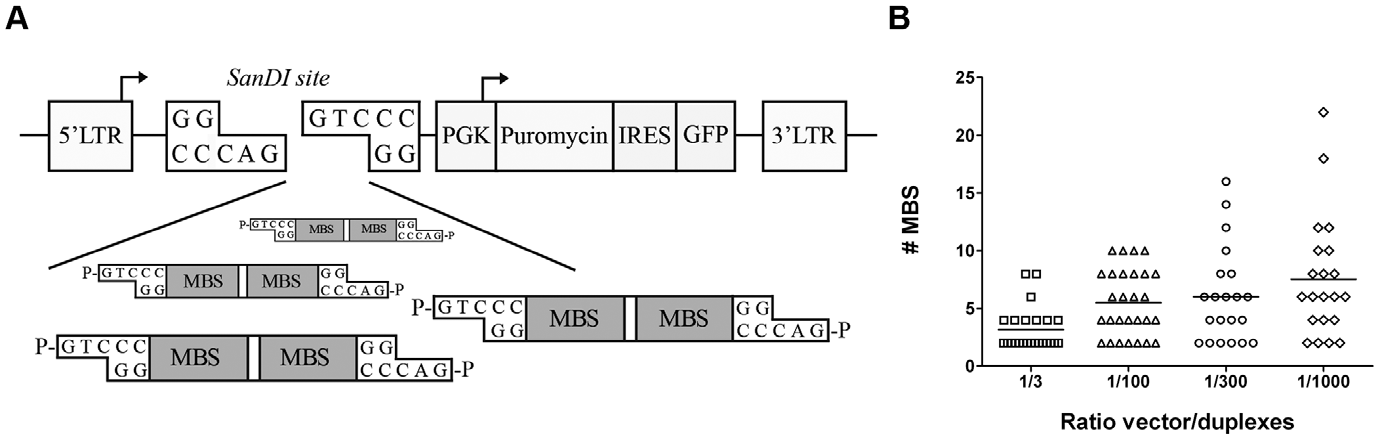
The rapid generation of miRNA sponges. (**A**) Schematic overview of the strategy to ligate miRNA sponge oligo duplexes into the pMSCV-PIG-sp vector. Each oligo duplex contains 2 miRNA binding sites (MBS) and phosphorylated SanDI restriction enzyme compatible overhangs to enable miRNA sponge generation in one single ligation reaction. SanDI overhangs are non-palindromic allowing for directional cloning of the oligo duplexes. Arrows indicate transcription start sites. (**B**) Overview of the number of MBS per clone generated from ligation reactions with vector/duplex ratios varying from 1∶3 up to 1∶1000. Per ratio the mean is indicated.

To enable directional cloning of the oligo duplexes we inserted a SanDI site in the pMSCV-PIG vector which will result in non-palindromic overhangs upon digestion. By ligating oligo duplexes with SanDI compatible ends with SanDI digested pMSCV-PIG-sp, sponge constructs with a variable number of MBS were generated in a single ligation reaction (Fig. 1a). This ligation strategy was performed with sponge oligo duplexes for miR-19 (bulged and perfect), miR-92a and miR-155 using vector to duplex ratios of 1∶3, 1∶100, 1∶300 and 1∶1000. The compiled result of the PCR based screening of in total 94 colonies is shown in Figure 1b. By increasing the ratio between vector and oligo duplexes from a 1∶3 ratio to a 1∶1000 ratio, the average number of MBS increased from 3.2 (range 2–8) to 7.5 (range 2–22). Within the 1∶1000 ratio ligation 29% of all analyzed clones had 10 or more MBS. Sanger sequencing of 10 clones with different inserts and insert lengths confirmed for all clones the expected number of MBS in the correct orientation. This shows that our method is a fast and efficient method allowing generation of miRNA sponges with a variable number of MBS.

To show that sponges generated by this method are fully functional, we performed several experiments. MiR-19 sponge variants containing 2–20 of either perfect or bulged MBS were used to test whether perfect or bulged MBS sponges are more effective. First we used the PITA and STarMir miRNA binding energy prediction algorithms to determine how favorable it is for miR-19 to bind to the miR-19 perfect and bulged MBS sponges compared to known proven endogenous miRNA-19 targets. Both algorithms predicted that miR-19a and miR-19b bind with a much lower ΔΔG/ΔG_total_ to the sponge MBS than to the MBS of seven proven endogenous targets (Fig. S1) [18], [19]. As expected, a lower ΔΔG/ΔG_total_ was calculated for the perfect MBS sponge as compared to the bulged MBS sponge.

**Figure 2:**
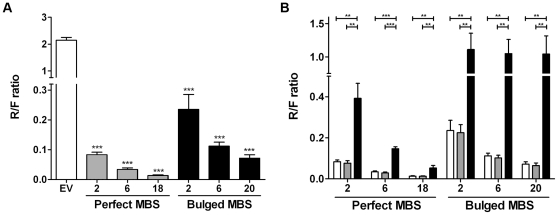
Confirmation of miR-19 binding to miR-19 sponge constructs. (**A**) Luciferase reporter assays in HEK293 cells reveal that repression of Renilla activity is more prominent in reporter vectors that contain perfect MBS sequences as compared to reporter vectors that encode bulged MBS sequences and is in both cases dependent on the number of MBS. (**B**) Release of miR-19 specific repression of Renilla luciferase activity by anti-miR-19a/b oligos confirms that miR-19 binds to the miR-19 MBS sequences. No release of luciferase activity is observed with a control anti-miR-16 oligo. Open bars: Mock, grey bars: miR-16 inhibitor and black bar: miR-19 inhibitor mix. For each graph the number of MBS per reporter vector is indicated on the x-axis and on the y-axis the ratio of Renilla (R) over Firefly (F) luciferase is depicted. * = p-value <0.05; ** = p-value <0.01; *** = p-value <0.001.

We confirmed binding of miR-19 to the MBS sequences by inserting these sequences in a luciferase reporter vector and perform luciferase based assays in HEK293 cells that endogenously express miR-19a and miR-19b at high levels (up to 10% of all miRNAs [20]). Compared to the empty vector, reporter vectors with 2 perfect MBS sequences showed >25 fold lower Renilla/Firefly (R/F) ratio while this was >9-fold for vectors with 2 bulged MBS sequences (Fig. 2a). Decreasing R/F ratios were observed with increasing number of MBS for both the perfect and bulged MBS containing reporter vectors. To confirm that the observed effects were indeed caused by binding of miR-19 to the MBS sequences, we cotransfected cells with miR-19 inhibitors or irrelevant miR-16 inhibitors and the reporter vector. This revealed that repression of Renilla activity could be efficiently prevented with miR-19 inhibitors but not with miR-16 inhibitors (Fig. 2b). Thus, both perfect and bulged MBS containing reporter vectors can efficiently bind miR-19 in a dose dependent way in a luciferase reporter assay. Prevention of repression of Renilla activity upon miR-19 inhibitor cotransfection was less effective in perfect MBS reporter vectors as compared to bulged MBS reporter vectors. One explanation for these differences is that they may be caused by degradation of Renilla transcripts with perfect MBS resulting in lower R/F ratios. Quantification of Renilla and Firefly transcripts by qRT-PCR revealed similar Renilla and Firefly transcript levels when comparing bulged and perfect MBS reporter constructs with the same number of MBS (Fig. S2). Equal firefly levels show that bulged and perfect reporters are expressed at equal levels and similar Renilla levels indicate that Renilla transcripts with perfect MBS do not seem to be subject to degradation. An alternative explanation for the observed difference in prevention of Renilla repression may be that the cotransfected miR-19 inhibitor oligos are less efficient in preventing miR-19 to bind to the perfect MBS sequences due to higher sequence complementarity.

**Figure 3:**
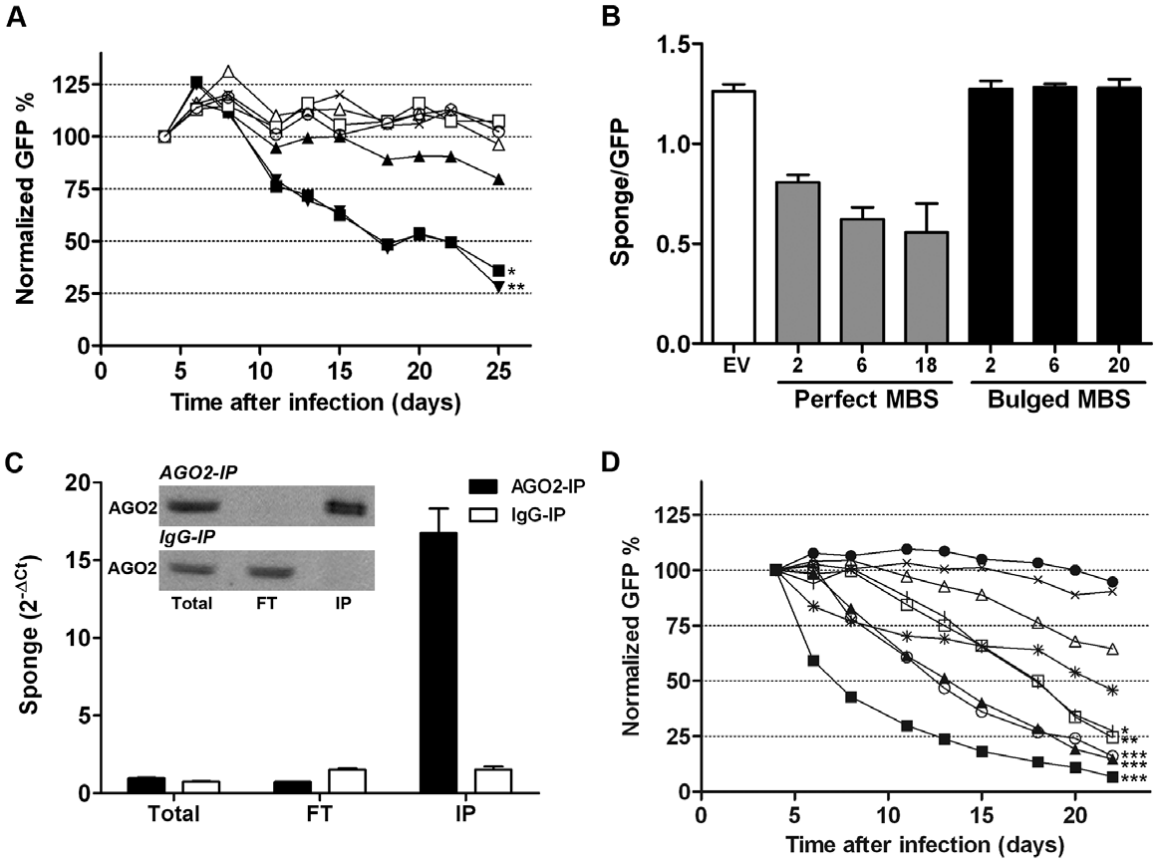
Bulged miRNA sponges can effectively inhibit miRNA function. (**A**) GFP competition assay in WEHI-231 B-cell lymphoma cells infected with empty vector (EV), bulged and perfect miR-19 sponges. Bulged MBS sponges have mild (2 MBS) or strong effects (6 and 20 MBS) on cell growth while perfect MBS sponges and the empty vector control have no effect. Legend: x = EV; Δ = P-2MBS; □ = P-6MBS; ○ = P-18MBS; ▴ = B-2MBS; ▪ =  = B-6MBS; ▾ = B-20MBS. (**B**) From the experiment shown in Fig. 3a, EV, perfect en bulged MBS sponge infected GFP+ cells were sorted at day 14. Quantification of sponge (LTR driven) and GFP transcript levels (PGK driven) by qRT-PCR revealed lowered sponge/GFP transcript ratios for the perfect sponges but not for the bulged sponges as compared to the empty vector control. The number of MBS per sponge vector is indicated on the x-axis. (**C**) Quantification of sponge transcript levels in the AGO2-immunoprecipitated (IP) fraction of miR-155 sponge infected KM-H2 cells. AGO2-IP of miR-155 sponge infected KM-H2 cells was performed and followed by quantification of miR-155 sponge transcript levels in total, flowthrough (FT) and IP fraction of AGO2 and IgG IP experiments. MiR-155 sponge transcripts are highly enriched in the AGO2-IP fraction but not in the IgG-IP control fraction. In the inset of the graph a Western Blot which shows that AGO2 levels are enriched in the IP fraction upon AGO2-IP but not in the IgG-IP control. (**D**) GFP competition assay in WEHI-231 cells infected with individual bulged miRNA sponges against each member of the miR-17∼92 cluster and combination (combi-sp) sponges against each miRNA family of the miR-17∼92 cluster. As a control, empty vector and a combi-sp vector with seed scrambled miRNA BS (combi-scr) were used. All individual miRNA sponges show negative effects on cell growth, however, the strongest effects are observed when all miRNAs of the miR-17∼92 cluster are inhibited simultaneously. No effects on proliferation were observed with the empty vector and combi-scr vector controls. Legend: x = EV; □ = miR-17-5p-sp (12MBS); Δ = miR-18a-sp (10MBS); * = miR-19-sp (20MBS); ı = miR-20a-sp (7MBS); ○ = miR-92a-sp (10MBS); ▪ = Combi-sp1 (4×3MBS); ▴ = Combi-sp2 (4×3MBS); • = Combi-scr (4×3MBSscr). * = p-value <0.05; ** = p-value <0.01; *** = p-value <0.001.

MiR-19 is part of the oncogenic miR-17∼92 cluster [21], [22] and has been shown to be crucial for the oncogenic properties of this cluster in a murine Myc-driven B-cell lymphoma model [19], [23]. Therefore, we determined to what extend our different miR-19 sponges could affect cell growth in murine WEHI-231 B-cell lymphoma cells which express miRNAs of the miR-17∼92 cluster at high levels (Fig. S3). WEHI-231 cells were infected with bulged and perfect miR-19 MBS sponge vectors and empty vector control and the percentage of GFP+ cells within the mixed cell population (30–60% infected cells 4 days after infection) was followed for 21 days. Inhibition of miR-19 with bulged MBS sponges could affect cell growth (Fig. 3a). The bulged sponge with 2 MBS showed a mild growth inhibitory effect. Sponges with 6 and 20 bulged MBS showed almost identical strong significant negative effects (p-value resp. <0.05 and <0.01) on cell growth with a 50% reduction in the percentage of GFP+ cells in 14 days. In contrast, no growth inhibition was observed for any of the perfect MBS sponges against miR-19. Despite similar initial infection percentages as determined by the percentage of GFP+ cells, the median fluorescence intensity (MFI) is on average 2-fold lower in perfect MBS sponges compared to EV and bulged MBS sponges (Fig. S4a and S4b). Since the luciferase assay proved that miR-19 can bind to the perfect MBS sponges we hypothesized that the lowered MFI observed for perfect sponges may be due to sponge transcript degradation as a result of perfect complementary miR-19 binding. Degradation of perfect MBS sponge containing Renilla transcripts was not observed in the luciferase experiments, however, this could be due to the fact that transcript levels were measured already 24 h after transfection (at the same time as the luciferase assay was performed). To test whether degradation could explain our observations we sorted cells infected with the bulged and perfect miR-19 MBS sponge vectors and empty vector control (Fig. 3a) based on GFP fluorescence two weeks after infection. Sponge transcript levels driven by the 5′LTR were quantified and compared to the GFP transcript levels which can be transcribed independently from the PGK promoter. This revealed that the ratio between sponge transcripts and GFP transcripts was lowered for perfect MBS sponges compared to that of the empty vector control, with the lowest ratio observed with 18 perfect MBS (>50% lower than empty vector, Fig. 3b and Fig. S5). In contrast, sponge/GFP transcript ratios for bulged sponges remained similar to empty vector control regardless of the number of MBS. Thus, our results show that bulged sponges are more effective for the prolonged inhibition of miRNAs than perfect sponges most likely (at least in part) due to degradation of the perfect MBS containing transcripts.

To further prove the functionality of our sponges we tested if the sponge transcripts, similar to endogenous miRNA targets, were enriched in the AGO2 immunoprecipitation (IP) fraction. Therefore, we infected the HL cell line KM-H2 that has high levels of miR-155 [24] with a miR-155 sponge (bulged, 14 MBS) and performed AGO2-IP. Quantitative RT-PCR showed that the sponge transcripts are indeed strongly enriched in the AGO2-IP fraction and not in the IgG control IP (Fig. 3c). Thus, similar to endogenous miRNA targets sponge transcripts are bound to AGO2 containing RISC complexes.

Next, we analyzed the effect of individual members of the miR-17∼92 cluster on WEHI-231 cells using bulged sponges. This revealed that all miRNAs of this cluster can individually influence WEHI-231 cell growth (Fig. 3d). A >50% reduction in GFP+ cells was observed within 18 days for all except the sponge construct against miR-18a which induced a 35% reduction of GFP+ cells. Almost identical curves were observed for the sponges that inhibit miR-17-5p and miR-20a, both members of the miR-17/106b seed family. No effect was observed for the empty vector control. To determine whether miRNAs of this cluster cooperatively act in the regulation of WEHI-231 growth we also designed 2 combination (combi)-sponges (combi-sp1 and -sp2). Both combi-sponges contained 3 MBS for each miRNA seed family of this cluster, i.e. miR-17, miR-18a, miR-19 and miR-92a, and only differed in the spacer sequences in between the MBS. PITA analyses predicted, based on ∼equal sum of ΔΔG, that both sponges would bind the corresponding miRNAs with equal efficiencies (Fig. S6). The STarMir algorithm predicted that binding of miRNAs of the miR-17∼92 cluster to combi-sp1 would be energetically more favorable than binding to combi-sp2 (∼2-fold lower sum of ΔG_total_ per miRNA). GFP competition assays with the combi-sponges revealed a strong decrease in cell growth, i.e. the percentage of GFP+ cells was halved within 3 and 9 days for respectively combi-sp1 and combi-sp2 (Fig. 3d). No effect on WEHI-231 cell growth was observed for the control combi-sponge which existed of the exact same nucleotide content as combi-sp1 with the miRNA seed binding regions for each BS being scrambled.

## Discussion

We developed a fast and efficient method to generate miRNA sponges with variable numbers of MBS. Sponges with more than ten MBS can be easily generated within days. This makes our method fast and cost effective compared to previously published methods. Our method can also be used to inhibit two miRNAs simultaneously by designing MBS for two different miRNAs within one oligo duplex. In case more than two different MBS are desired it may be more efficient to design the sponges as minigenes. Drawbacks of minigenes are their costs and difficulties in synthesis that can be encountered with repeated sequences as present in sponges. Using various assays we showed that sponges generated using our method are fully functional. Firstly, luciferase assays confirm that miRNAs can bind to the sponges in a dose dependent manner. Secondly, GFP assays show that the sponges can functionally inhibit miRNAs. Thirdly, AGO2-IP showed that the sponges are enriched in the AGO2-IP fraction, similar to endogenous targets.

Based on the long-term GFP competition assay with various miR-19 sponges it is clear that miRNA sponges with bulged MBS effectively inhibit miRNAs while sponges with perfect MBS do not. A limited effect on cell growth was observed using two bulged MBS and similar strong effects were observed using sponge constructs containing six and twenty MBS. Thus, in WEHI-231 cells introduction of miR-19 sponges with six bulged MBS already suffices for maximal miRNA inhibition. Depending on the level of miRNA expression and the number and binding energy of endogenous miRNA targets more or less MBS may suffice for maximal miRNA inhibition [17]. In the case of miR-17 inhibition in WEHI-231, sponges with twelve MBS showed a 2-fold faster reduction in GFP+ cells as compared to sponges with four MBS (data not shown). No effects on cell growth were observed for perfect MBS sponges regardless the number of MBS incorporated in the sponge construct. The lower effectiveness of perfect sponges compared to bulged sponges can at least in part be explained by the lower perfect sponge RNA transcripts levels. It has been reported that upon perfect miRNA binding, mRNAs are cleaved by the slicer activity of the RISC complex [13]. Thus, although we do not show direct cleavage of perfect MBS sponge transcripts it is likely that the perfect MBS sponge transcript levels are lowered due to mRNA cleavage and therefore are less suitable for miRNA inhibition. Nevertheless, perfect MBS sequences are suitable for miRNA mediated downregulation of a specific transcript [25], [26] as we show in our luciferase reporter assay. Both PITA and STarMir predicted a more efficient binding of miRNAs to perfect MBS sponges compared to bulged MBS sponges consistent with the results obtained from the luciferase assay. However, neither PITA nor STarMir take into consideration the effects of sponge degradation upon binding of a perfectly matched antisense miRNA. Thus, when designing sponges for long-term miRNA inhibition one should not solely focus on the ΔΔG/ΔG_total_ but also ensure that MBS are not perfect antisense.

Finally, we successfully used bulged sponges against each of the miRNAs of the miR-17∼92 cluster to determine the effect on cell growth of each member in a GFP competition assay (Fig. 3d). This revealed that inhibition of miR-92 was most effective for growth inhibition followed by miR-17 and miR-20, miR-19 and miR-18 being the least effective. Elegant studies in a mouse model of B-cell lymphoma showed that miR-19a and miR-19b were required and largely sufficient for the oncogenic properties of the miR-17∼92 cluster [19], [23]. However, in WEHI-231 cells the twelve MBS sponge against miR-92a resulted in a >2× faster reduction in GFP+ cells than a twenty MBS sponge against miR-19. It is unlikely that this is due to incomplete sequestration of miR-19 since sponges with six and twenty MBS showed similar effects in the GFP competition assay indicating maximal miRNA inhibition (Fig. 3a). Thus, our results suggest that in WEHI-231 miR-92a has a more prominent role in regulating cell growth than miR-19. The MBS of the miR-17 and miR-20 sponges differ at nt 1, 14 and 22, the sequence between the MBS, and the number of MBS (resp. 12 and 7 BS). Nonetheless, the sponges against miR-17 and miR-20 are both approximately equally effective in growth inhibition in the GFP competition assay. This is in line with the idea that sponges can inhibit all miRNAs with the same seed sequence. The two combination sponges against the entire miR-17∼92 revealed a 50% reduction of GFP+ cells after 3 and 9 days for combi-sp1 and combi-sp2 respectively. This denotes cooperation of the individual miR-17∼92 cluster members in promoting proliferation/oncogenic activity. The difference between combi-sp-1 and combi-sp-2 is in the sequence of the 4 nt spacers between the MBS. As a result, combi-sp-1 is predicted by STarMir to sequester miRNAs more efficiently than combi-sp-2 which is consistent with the faster reduction in GFP+ cells for combi-sp1.

In conclusion, we have developed a fast and efficient method to generate miRNA sponges that can be applied to stably inhibit one or more miRNAs. Luciferase, GFP competition and AGO2-IP assays show that the sponges generated using our protocol are efficient inhibitors of miRNAs. Our method will prove to be highly valuable for studies that involve generating miRNA sponges to study miRNA loss of function either *in vitro* or *in vivo*.

## Materials and Methods

### Cell lines

The Hodgkin lymphoma cell line KM-H2 was cultured in RPMI 1640 supplemented with 2 mM ultra-glutamine, 100 U/ml penicillin/streptomycin, and 10% fetal bovine serum (Cambrex Biosciences, Walkersville, USA). For the murine WEHI-231 cell line, 50 µM 2-mercaptoethanol was added as an extra additive. Phoenix-ampho [27], HEK-293 and HEK-293T cells were cultured in DMEM supplemented with 2 mM ultraglutamine, 100 U/ml penicillin/streptomycin, and 10% fetal bovine serum (Cambrex Biosciences). All cell lines except Phoenix-ampho were obtained from the DSMZ (KM-H2, HEK-293 and HEK-293T) or ATCC (WEHI-231).

### Individual miRNA sponge design

To enable directional cloning of repetitive miRNA BS sequences, we designed oligo duplexes with overhangs that are compatible with the restriction endonuclease SanDI [28]. This enzyme recognizes the 7-bp interrupted palindrome 5′-GGGWCCC-3′ (W = A or T) producing 3-nt long 5′ protruding (GWC) that will enable directional ligation of the oligo duplexes. Besides the phosphorylated SanDI compatible overhangs each oligo duplex contained 2 MBS separated by a 4 nt spacer sequence that were either perfect (-P) antisense to the miRNA or contained a central 4 nt mismatch (“bulge”, -B) to the miRNA. To optimize the sequence of our sponges, two published algorithms, i.e. PITA (http://genie.weizmann.ac.il/pubs/mir07/mir07_prediction.html, [29]) and STarMir (http://sfold.wadsworth.org/cgi-bin/STarMir.pl were used with standard settings [30]). For the *in silico* analyses, sponge sequence and SanDI site flanking sequence starting from the end of the psi packaging signal and ending at the start of the PGK promoter of the pMSCV-PIG-sp vector (see below) were used (total of 466 bp+sponge sequence). Based on different RNA folding computations, both algorithms predict the effectiveness of the designed MBS in the sponges by calculating the difference between the free energy gained by binding of the miRNA to the MBS and the free energy lost by unwinding of the MBS nucleotides (ΔΔG (PITA) and ΔG_total_ (STarMiR)). The PITA algorithm also provides information on all other miRNAs that can potentially bind to the sponge sequence. This information was used to vary the sequence to minimize off target miRNA binding. Variations that were tested included varying the sequence and length of the spacer sequence (sequence between to MBS) and switching between A and T nucleotides in the middle of the SanDI recognition sequence. Bulged MBS sponges were generated for miR-19, miR-92a and miR-155. For miR-19 we also created perfect sponges. The miR-19 family consists of miR-19a and -19b that differ at nt 11 (U/C). To allow perfect antisense binding of miR-19a as well as miR-19b we designed the perfect sponges to have an equal number of MBS against miR-19a and -19b (Table S1 and S2). The bulged miR-19 sponge contained identical MBS for miR-19a and miR-19b since the differing nt 11 was within the central bulge.

### MicroRNA combination sponges

Combination sponges for the miR-17∼92 cluster containing 3 MBS for the miR-17, miR-18a, miR-19 and miR-92 family were ordered as minigenes (IDT, Coralville, IO, USA) with 5′-Xho1 and 3′-EcoR1 sites for subcloning into pMSCV-PIG-sp (combi-sp1 and –sp2, full sequences in Table S2). For optimal design of the minigenes, PITA and STarMir were used to predict which variations in the spacer sequences between the MBS would result in maximal MBS affinity. Two versions of the miR-17∼92 sponge were designed that differed in the spacer sequences (combi-sp1 -TTAT- and combi-sp2 -GCGG- and -CGCC-). As a control, a multisponge with scrambled miRNA seed binding regions was designed (“combi-scr”, -TTAT- spacers).

### MicroRNA sponge generation

MBS containing oligos with 5′phosphates (Table S1) were ordered PAGE purified at a 100 nmol scale (IDT). Oligos were dissolved to 50 µM in STE^−4^ (100 mM NaCl, 10 mM Tris/HCL, 1 mM EDTA, pH 8.0). Sense and antisense oligos were mixed at a 1∶1 ratio and annealed by incubation at 100°C for 10 minutes followed by slow cooling. The pMSCV-PIG-sp vector was generated by inserting a SanD1 restriction site containing linker (Table S1) between the XhoI and EcoRI restriction sites of pMSCV-PIG (Addgene, www.addgene.org, plasmid 21654). pMSCV-PIG-sp was digested with SanD1 for 1 h at 37°C (Fermentas, St. Leon-Rot, Germany) and dephosphorylated with 1 µl CIAP (1 U/ul, Invitrogen, Carlsbad, CA, USA) for 5 minutes at 37°C. Enzymes were inactivated by incubation for 10 minutes at 65°C. Ligation was performed with 50–100 ng gel-purified vector and insert was added in different vector/oligo duplex ratios varying from 1∶3 to 1∶1,000. The amount of insert needed per ratio was calculated according to the formula: ng oligo duplex = {(size oligo duplex×ng vector)/size vector}×ratio. Ligation reactions were performed in 20 µl with 5 U T4 DNA ligase (Invitrogen). To determine the insert size, 10–20 colonies were screened by PCR after transformation (for primers see Table S1). A random selection of the constructs containing 1–10 oligo duplexes were subjected to Sanger sequencing for validation (LGC Genomics, Berlin, Germany). A sponge for miR-17-5p was generated using a previously described protocol [5] (for full sponge sequences see Table S2). Sponges for miR-18a and miR-20a (a kind gift from M. Ebert, Koch Institute for Integrative Cancer Research, MIT, USA) were subcloned into pMSCV-PIG-sp using XhoI and PmeI.

### Luciferase assay

MiR-19 sponge sequences were subcloned from the pMSCV-PIG-sp vector into the psiCHECK2 vector (Promega, Madison, USA) using Xho-1 and Pme-1 restriction enzymes. Luciferase reporter assays were performed as described previously [31]. Briefly, transfection of HEK293 cells was performed using the Amaxa nucleofector I device (Amaxa, Gaithersburg, USA) with solution V, program Q-01. One million HEK293 cells transfected with 2 µg of each construct with or without a 2 nmol equal molar mix of miR-19a and miR-19b inhibitor oligos or miR-16 inhibitor as a control (for sequence and modifications see Table S1, Exiqon, Vedbaek, Denmark) were harvested 24 h post transfection for dual luciferase measurement. For each transfection the Renilla and Firefly activity was measured in duplo and the average Renilla over Firefly (R/L) ratio was calculated. All transfections were performed in triplicate, and standard deviations were calculated. Repeated measures ANOVA followed by a Dunnett's multiple comparison post test was performed for each construct to determine whether there were significant differences in R/L ratios between the empty vector and the MBS containing reporter vectors. To calculate whether mock, miR-19 inhibitor or miR-16 inhibitor treatment significantly altered the R/L ratio of a reporter vector we performed repeated measures ANOVA followed by Tukey's multiple comparison post test.

### Retroviral infection

For the production of ecotropic retrovirus, 293 T cells were CaPO_4_ transfected with 2.5 µg of retroviral expression vector and 2.5 µg pCL-Eco vector in 6 well format and virus was harvested after 48 h. For the generation of amphotropic retrovirus, Phoenix-Ampho cells were transfected with 37.5 µg vector in T75 flasks. Virus was harvested after 2 days and concentrated with Retro-X concentrator (Clontech, Saint-Germain-en-Laye, France) according to the manufacturer's protocol. For both pseudotyped viruses target cells were infected by spinning at 2000 rpm with a final polybrene concentration of 4 µg/ml for 2 hrs.

### FACS analysis

For GFP competition assays WEHI-231 cells were freshly infected (infection rate 25–70%, adjusted to max 50% GFP+ cells) and after 4 days GFP expression was monitored for three weeks tri-weekly. Data were acquired on a FACS Calibur flow cytometer (BD PharMingen) and analyzed using FlowJo software (version 7.6, Treestar, Ashland, OR). The GFP percentage determined at the first day of measurement (4 days post infection) was set to 1. For each measurement the average decline per day was calculated and used to determine whether the average decline in GFP percentage was significant different from the empty vector control (repeated measures one-way ANOVA, with Dunnet's multiple comparison post test, p-value <0.05). Each GFP competition assay was performed at least twice. For qRT-PCR and AGO2-IP experiments infected cells were GFP sorted ∼2 weeks after infection using a MoFlo sorter (Dako cytomation).

### qRT-PCR analysis

Total RNA was isolated using Trizol (Invitrogen, Carlsbad, CA). cDNA was made with 500 ng input RNA using SuperScript II and random hexamers according to the manufacturers protocol (Invitrogen). Quantitative PCR (qPCR) was performed using primers that amplify a 56 bp region 3′of the SanDI restriction site resulting in specific detection of the sponge transcript made from the 5′LTR and primers that amplify a 57 bp region within the GFP transcript that can detect the PGK promoter driven transcript and possibly “read-through” transcripts from the 5′LTR (for schematic overview of the used vector see Fig. 1a and for primer sequences see Table S1). qPCR was performed in triplicate in 20 µl reaction volumes containing either 1× SYBRgreen mix (Applied Biosystems, Foster City, CA, USA), 300 nM primers, and 2 ng of cDNA (for sponge and GFP transcript) or 1× PCR mix (Eurogentec, Maastricht, The Netherlands) 900 nM pimers and 500 nM probe (for U6). Reactions were performed on an ABI7900HT Sequence Detection System device (Applied Biosystems) using the standard program. Mean cycle threshold (C_t_) values for all genes were quantified with the SDS software (Applied Biosystems; version 2.1). Relative expression levels of sponge and GFP transcripts in WEHI-231 cells were calculated by normalizing expression levels to U6 resulting in a ΔC_t_ from which the 2^−ΔCt^ was calculated. For the quantification of Renilla and Firefly luciferase transcripts in HEK293 cells relative expression levels were calculated by normalizing to RPII. For the quantification of the miR-155 sponge transcript levels in the AGO2-IP analysis, Ct values were corrected to equalize for the input of each fraction. qRT-PCR for miRNA-17-5p, -18a, -19a, -19b, -20a and -92a were performed using miRNA qRT-PCR assays (Applied Biosystems) according to the manufacturer's protocol. MiRNA expression levels were normalized to the levels of snoRNA-429.

### AGO2-immunoprecipitation and Western blot

Immunoprecipitation of AGO2-containing RISC complexes was performed as published previously [32]. Briefly, 20 million miR-155 sponge or empty vector infected sorted cells were lysed. Cleared supernatant was incubated with protein G sepharose beads coated with AGO2 antibody (Clone 2E12-1C9, Abnova, Taiwan) or IgG control antibody at 4°C overnight. After washing of the beads, RNA was harvested for qRT-PCR analysis and protein lysates were made for Western blot analysis. AGO2 Western blotting was performed as described previously [32].

## Supporting Information

Figure S1 **Pita and STarMir analyses for miR-19 sponge variants and seven proven miR-19 targets.** Both algorithms calculate the difference between the amount of energy needed to make the MBS available for binding and the amount of energy that is gained by base pairing of the miRNA to the MBS (ΔΔG (PITA) and ΔG_total_ (STarMir), Y-axis). The predicted MBS within the sponge sequence or within the 3′UTR of endogenous targets are ordered from low to high ΔΔG/ΔG_total_. The majority of MBS present in the miR-19 sponges are predicted to have a much lower ΔΔG/ΔG_total_ than MBS in proven endogenous miR-19 targets. Note 1: perfect BS sponges in the PITA analysis shows 12 MBS instead of the expected 6 MBS due to the fact that nt 3–9 of miR-19a and miR-19b are repeated at nt 13–19 and are therefore in the perfect antisense sequence defined by PITA as potential MBS; Note 2: no miR-19b MBS were predicted by STarMir for SBF2.
(TIF)

Figure S2 **Renilla and Firefly luciferase quantification.** RNA was isolated from HEK293 cells, 24 h after transfection of the luciferase reporter vector. Renilla (top) and Firefly (middle) transcript levels were quantified to show that perfect and bulged MBS reporter vectors with the same number of MBS are expressed at similar levels. Renilla transcript levels normalized to those for Firefly revealed no evidence for increased degradation of Renilla transcripts in perfect MBS sponges as compared to bulged MBS sponges (bottom).
(TIF)

Figure S3 **Quantification of miRNAs of the miR-17∼92 cluster in WEHI-231 cells.** All miRNA levels of the miR-17∼92 cluster miRNAs were quantified and normalized to snoRNA429. Lower levels were observed for miR-18a, intermediate levels for miR-17-5p, miR-19a, miR-19b and miR-20a and higher levels for miR-92a.
(TIF)

Figure S4 **Median fluorescence intensity (MFI) is lower in perfect MBS sponges as compared to bulged MBS sponges and empty vector at similar infection rates.** (**A**) GFP percentages at day 4 after infection of empty vector (EV, open bars) and perfect (striped bars) and bulged (grey bars) sponges with indicated amounts of MBS. (**B**) MFI for the same constructs at the same time point. For each graph the number of MBS per sponge vector is indicated on the x-axis.
(TIF)

Figure S5 **Perfect MBS sponge transcripts levels are lower than bulged sponge transcript levels.** Two weeks after infection WEHI-231 cells infected with miR-19 perfect and bulged MBS sponges were sorted and RNA was isolated (experiment shown in Fig. 3a). Sponge transcript (LTR driven, top) quantification revealed that lower levels of sponge transcripts are present in cells infected with the perfect MBS sponges compared to bulged MBS sponges. GFP transcript levels (PGK driven and not regulated by miR-19, middle) are similar for 6 MBS and 18/20 MBS sponges. The 2 MBS sponges showed a ∼2,5 fold difference in GFP transcript levels between bulged and perfect MBS sponges. Sponge transcript levels normalized to GFP revealed that sponge transcript levels are consistently lower in perfect MBS sponges compared to bulged MBS sponges and the empty vector control (bottom).
(TIF)

Figure S6 **Pita and STarMir analyses for combi-sp1 and combi-sp2 sponges.** For each miRNA of the miR-17∼92 cluster, i.e. miR-17, miR-18a, miR-19a, miR-19b, miR-20a and miR-92a, the sum of the ΔΔG/ΔG_total_ of all MBS is calculated. As a control the combi-scr was also analyzed for predicted miRNA binding. (**A**) The PITA algorithm predicts that binding of miR-17∼92 miRNAs to combi-sp1 or combi-sp2 is∼equally energetically favorable. (**B**) The STarMir algorithm predicts that miRNAs of the cluster bind with a lower ΔG_total_ to combi-sp1.
(TIF)

Table S1 **Oligos used.**
(XLS)

Table S2 **Sponge sequences.**
(DOC)
