# Supplementary figures and images for: Rapid Generation of MicroRNA Sponges for MicroRNA Inhibition
Source: PLoS One. 2012 Jan 6;7(1):e29275. doi: 10.1371/journal.pone.0029275 (PMC3253070; doi:10.1371/journal.pone.0029275)

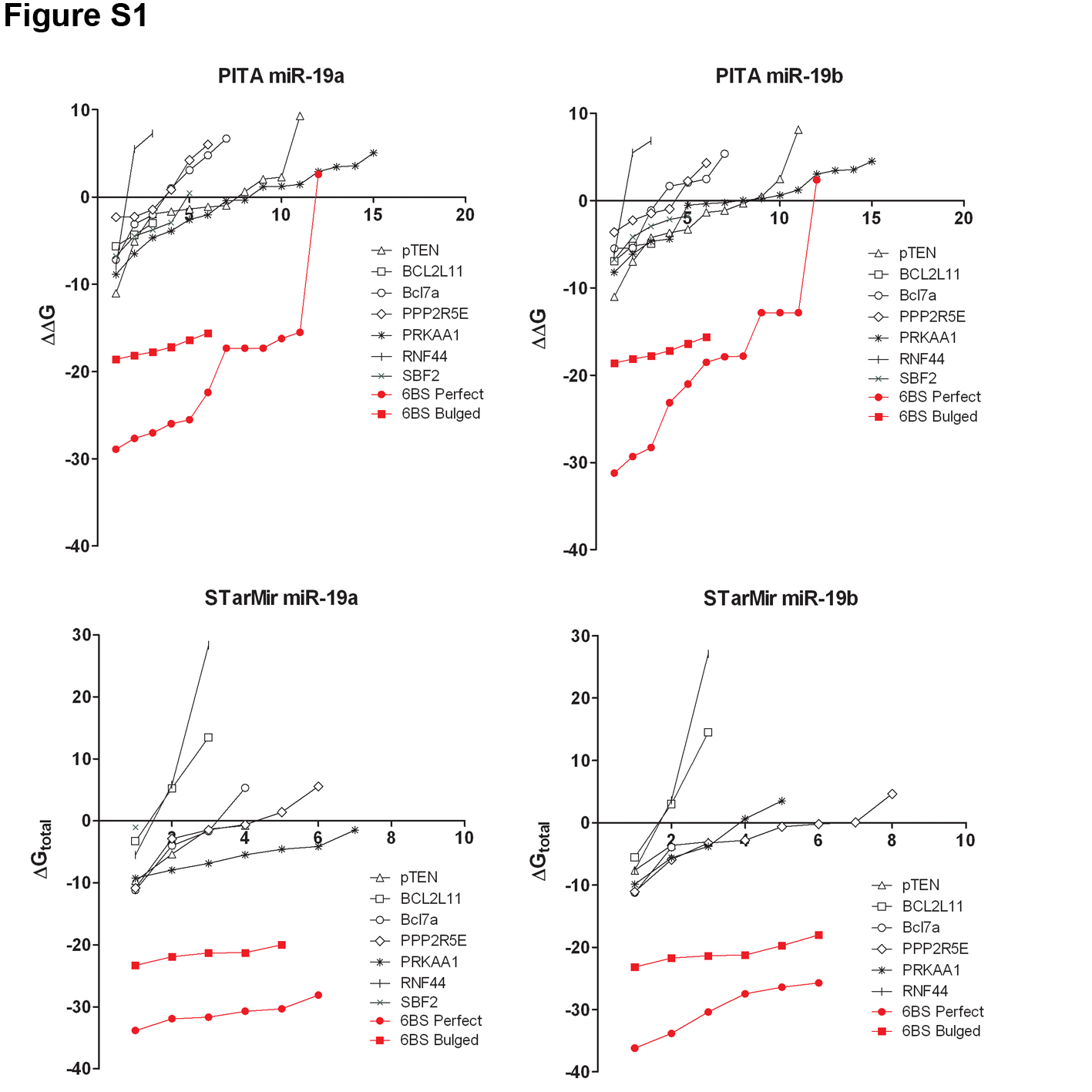

Supplement: Figure S1 — Pita and STarMir analyses for miR-19 sponge variants and seven proven miR-19 targets. Both algorithms calculate the difference between the amount of energy needed to make the MBS available for binding and the amount of energy that is gained by base pairing of the miRNA to the MBS (ΔΔG (PITA) and ΔGtotal (STarMir), Y-axis). The predicted MBS within the sponge sequence or within the 3′UTR of endogenous targets are ordered from low to high ΔΔG/ΔGtotal. The majority of MBS present in the miR-19 sponges are predicted to have a much lower ΔΔG/ΔGtotal than MBS in proven endogenous miR-19 targets. Note 1: perfect BS sponges in the PITA analysis shows 12 MBS instead of the expected 6 MBS due to the fact that nt 3–9 of miR-19a and miR-19b are repeated at nt 13–19 and are therefore in the perfect antisense sequence defined by PITA as potential MBS; Note 2: no miR-19b MBS were predicted by STarMir for SBF2. (TIF) [file pone.0029275.s001.tif]

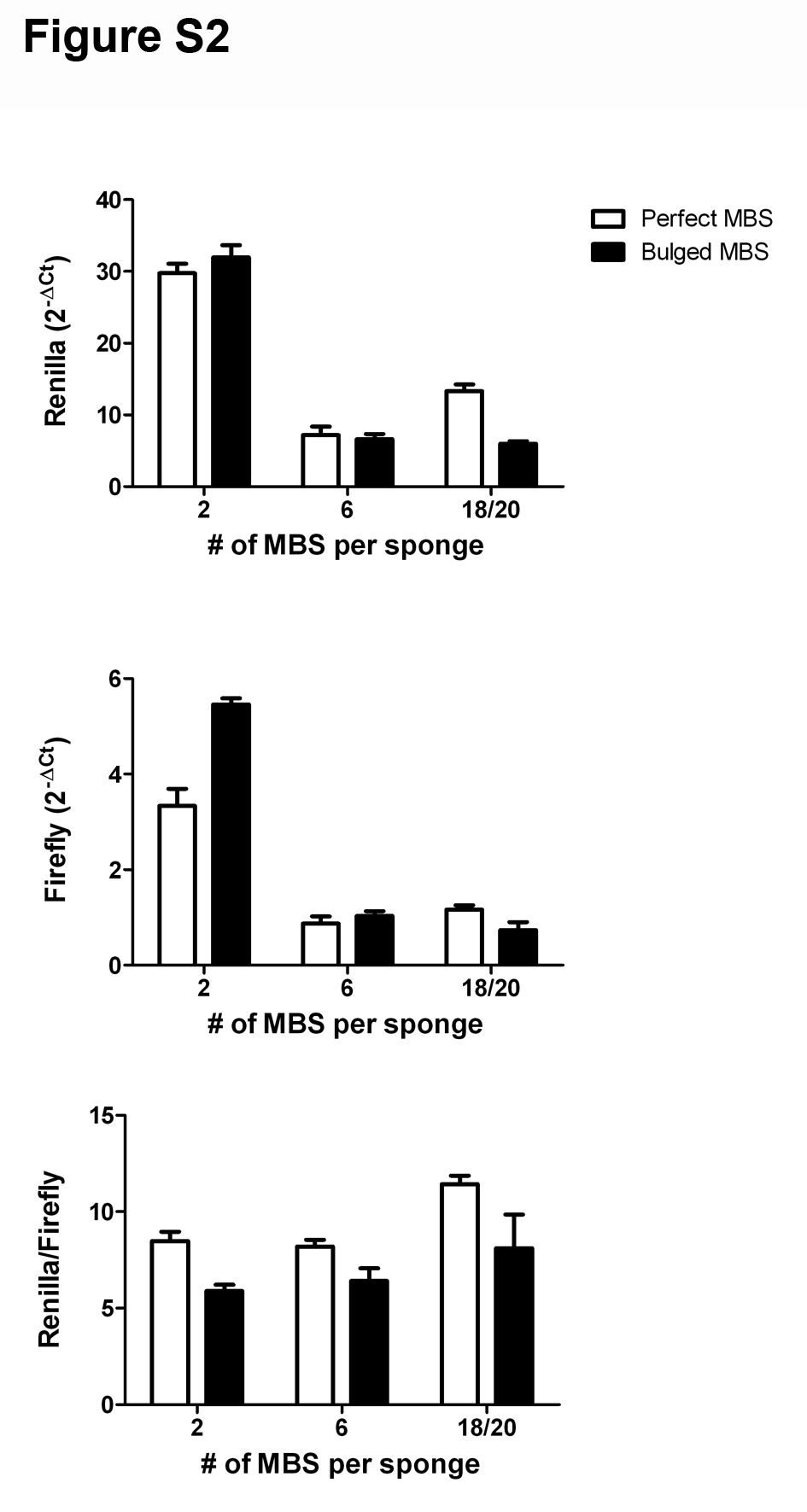

Supplement: Figure S2 — Renilla and Firefly luciferase quantification. RNA was isolated from HEK293 cells, 24 h after transfection of the luciferase reporter vector. Renilla (top) and Firefly (middle) transcript levels were quantified to show that perfect and bulged MBS reporter vectors with the same number of MBS are expressed at similar levels. Renilla transcript levels normalized to those for Firefly revealed no evidence for increased degradation of Renilla transcripts in perfect MBS sponges as compared to bulged MBS sponges (bottom). (TIF) [file pone.0029275.s002.tif]

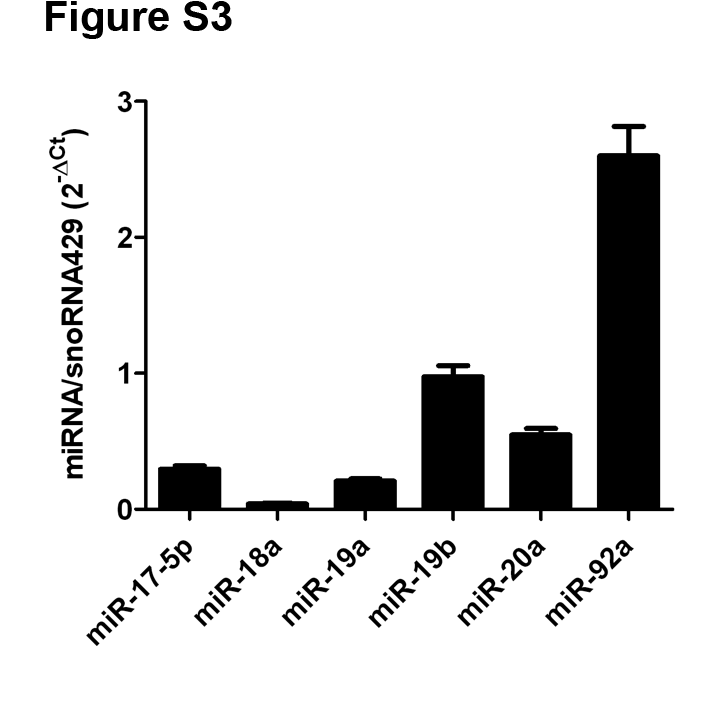

Supplement: Figure S3 — Quantification of miRNAs of the miR-17∼92 cluster in WEHI-231 cells. All miRNA levels of the miR-17∼92 cluster miRNAs were quantified and normalized to snoRNA429. Lower levels were observed for miR-18a, intermediate levels for miR-17-5p, miR-19a, miR-19b and miR-20a and higher levels for miR-92a. (TIF) [file pone.0029275.s003.tif]

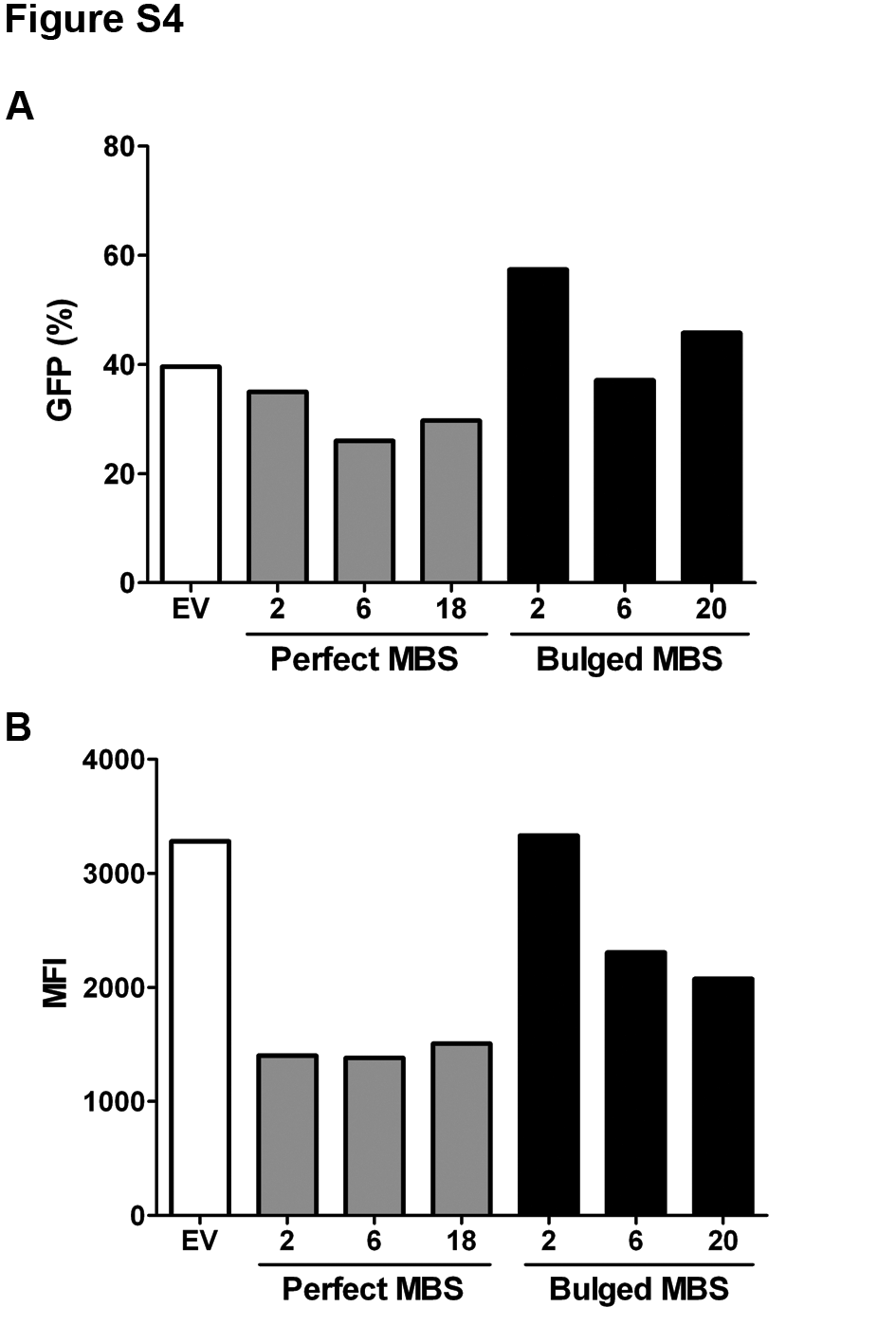

Supplement: Figure S4 — Median fluorescence intensity (MFI) is lower in perfect MBS sponges as compared to bulged MBS sponges and empty vector at similar infection rates. (A) GFP percentages at day 4 after infection of empty vector (EV, open bars) and perfect (striped bars) and bulged (grey bars) sponges with indicated amounts of MBS. (B) MFI for the same constructs at the same time point. For each graph the number of MBS per sponge vector is indicated on the x-axis. (TIF) [file pone.0029275.s004.tif]

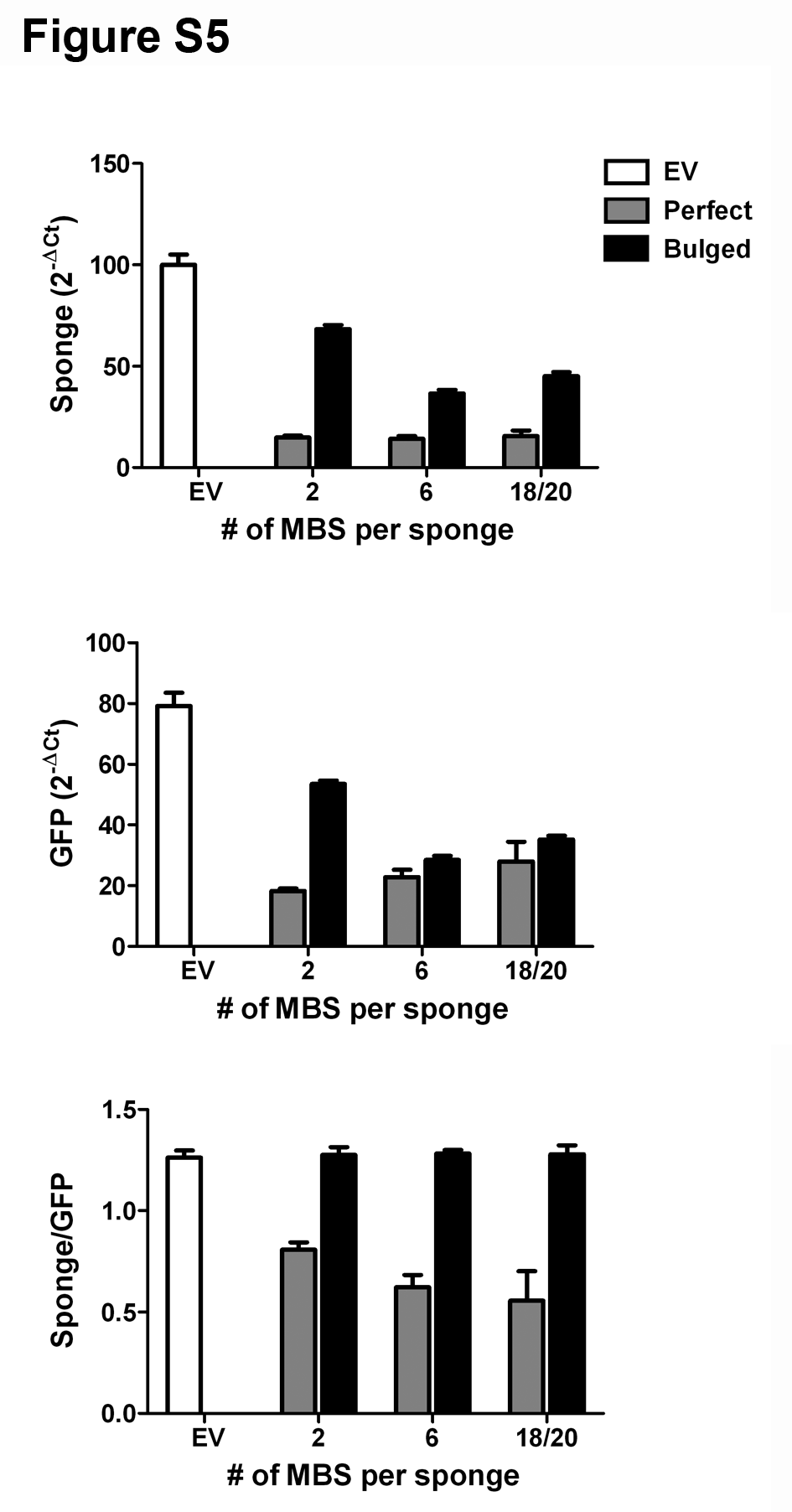

Supplement: Figure S5 — Perfect MBS sponge transcripts levels are lower than bulged sponge transcript levels. Two weeks after infection WEHI-231 cells infected with miR-19 perfect and bulged MBS sponges were sorted and RNA was isolated (experiment shown in Fig. 3a). Sponge transcript (LTR driven, top) quantification revealed that lower levels of sponge transcripts are present in cells infected with the perfect MBS sponges compared to bulged MBS sponges. GFP transcript levels (PGK driven and not regulated by miR-19, middle) are similar for 6 MBS and 18/20 MBS sponges. The 2 MBS sponges showed a ∼2,5 fold difference in GFP transcript levels between bulged and perfect MBS sponges. Sponge transcript levels normalized to GFP revealed that sponge transcript levels are consistently lower in perfect MBS sponges compared to bulged MBS sponges and the empty vector control (bottom). (TIF) [file pone.0029275.s005.tif]

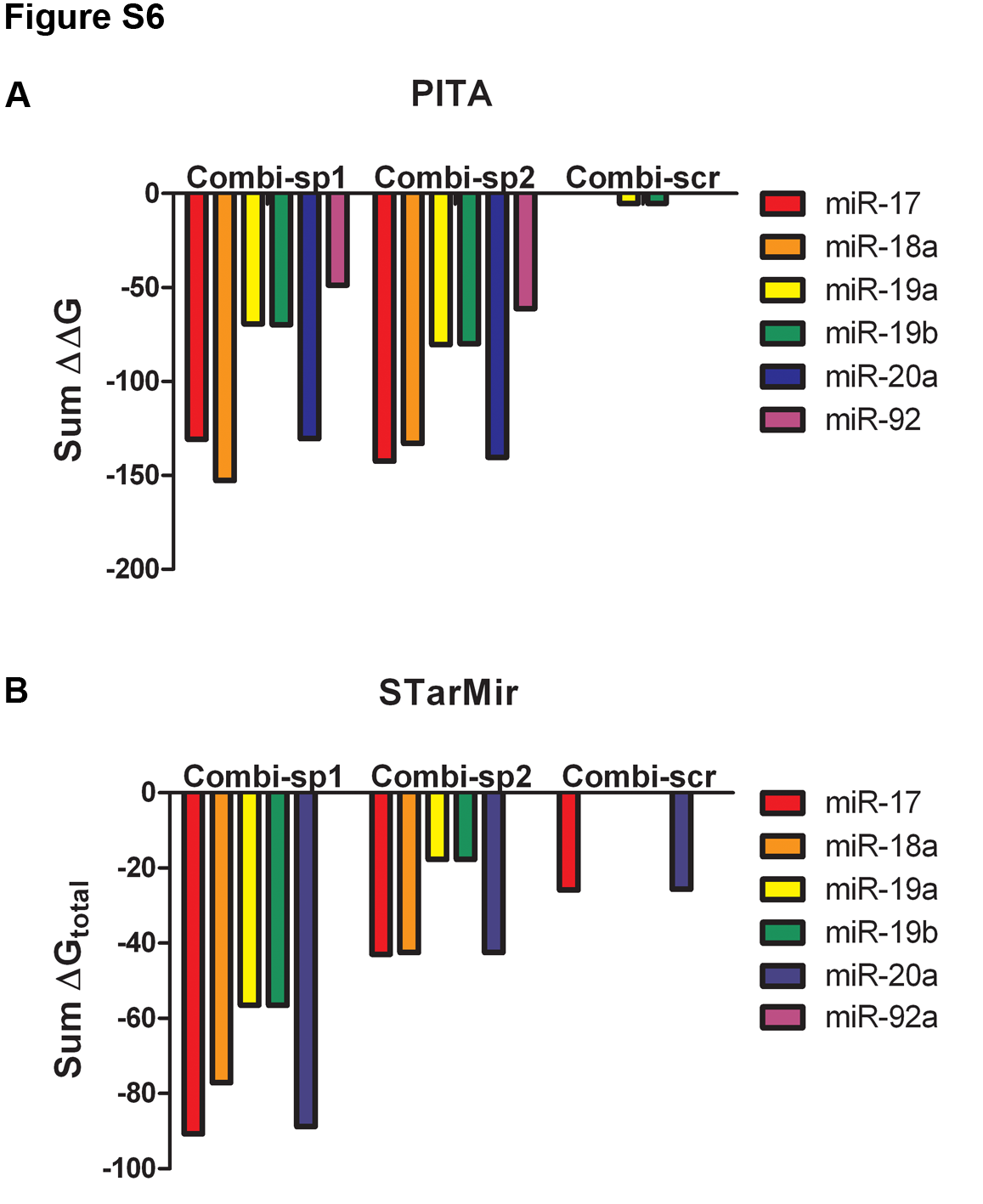

Supplement: Figure S6 — Pita and STarMir analyses for combi-sp1 and combi-sp2 sponges. For each miRNA of the miR-17∼92 cluster, i.e. miR-17, miR-18a, miR-19a, miR-19b, miR-20a and miR-92a, the sum of the ΔΔG/ΔGtotal of all MBS is calculated. As a control the combi-scr was also analyzed for predicted miRNA binding. (A) The PITA algorithm predicts that binding of miR-17∼92 miRNAs to combi-sp1 or combi-sp2 is∼equally energetically favorable. (B) The STarMir algorithm predicts that miRNAs of the cluster bind with a lower ΔGtotal to combi-sp1. (TIF) [file pone.0029275.s006.tif]
