# Supplementary material for: Rapid Generation of MicroRNA Sponges for MicroRNA Inhibition
Source: PLoS One. 2012 Jan 6;7(1):e29275. doi: 10.1371/journal.pone.0029275 (PMC3253070; doi:10.1371/journal.pone.0029275)
Supplement: Table S2 — Sponge sequences. (DOC) [file pone.0029275.s008.doc]

| **Table S2. Sequences of the used miRNA sponges.** | |
| --- | --- |
| Name | Sequence (from 5’-XhoI to EcoRI-3’ or PmeI-3’) |
| miR-19-P sponge  (2xMBS) | *CTCGAG*CTGGTTAACGACGGGTCCC**TCAGTTTTGCATAGATTTGCACA**AATT**TCAGTTTTGCATGGATTTGCACA**GGGTCCCGACGTTTAAACGAC*GAATTC* |
| miR-19-P sponge  (6xMBS) | *CTCGAG*CTGGTTAACGACGGGTCCC**TCAGTTTTGCATAGATTTGCACA**AATT**TCAGTTTTGCATGGATTTGCACA**GGGTCCC**TCAGTTTTGCATAGATTTGCACA**AATT**TCAGTTTTGCATGGATTTGCACA**GGGTCCC**TCAGTTTTGCATAGATTTGCACA**AATT**TCAGTTTTGCATGGATTTGCACA**GGGTCCCGACGTTTAAACGAC*GAATTC* |
| miR-19-P sponge  (18xMBS) | *CTCGAG*CTGGTTAACGACGGGTCCC**TCAGTTTTGCATAGATTTGCACA**AATT**TCAGTTTTGCATGGATTTGCACA**GGGTCCC**TCAGTTTTGCATAGATTTGCACA**AATT**TCAGTTTTGCATGGATTTGCACA**GGGTCCC**TCAGTTTTGCATAGATTTGCACA**AATT**TCAGTTTTGCATGGATTTGCACA**GGGTCCC**TCAGTTTTGCATAGATTTGCACA**AATT**TCAGTTTTGCATGGATTTGCACA**GGGTCCC**TCAGTTTTGCATAGATTTGCACA**AATT**TCAGTTTTGCATGGATTTGCACA**GGGTCCC**TCAGTTTTGCATAGATTTGCACA**AATT**TCAGTTTTGCATGGATTTGCACA**GGGTCCC**TCAGTTTTGCATAGATTTGCACA**AATT**TCAGTTTTGCATGGATTTGCACA**GGGTCCC**TCAGTTTTGCATAGATTTGCACA**AATT**TCAGTTTTGCATGGATTTGCACA**GGGTCCC**TCAGTTTTGCATAGATTTGCACA**AATT**TCAGTTTTGCATGGATTTGCACA**GGGTCCCGACGTTTAAACGAC*GAATTC* |
| miR-19-B sponge  (2xMBS) | *CTCGAG*CTGGTTAACGACGGGTCCC**TCAGTTTTGCCCTATTTGCACA**AATT**TCAGTTTTGCCCTATTTGCACA**GGGTCCCGACGTTTAAACGAC*GAATTC* |
| miR-19-B sponge  (6xMBS) | *CTCGAG*CTGGTTAACGACGGGTCCC**TCAGTTTTGCCCTATTTGCACA**AATT**TCAGTTTTGCCCTATTTGCACA**GGGTCCC**TCAGTTTTGCCCTATTTGCACA**AATT**TCAGTTTTGCCCTATTTGCACA**GGGTCCC**TCAGTTTTGCCCTATTTGCACA**AATT**TCAGTTTTGCCCTATTTGCACA**GGGTCCCGACGTTTAAACGAC*GAATTC* |
| miR-19-B sponge  (20xMBS) | *CTCGAG*CTGGTTAACGACGGGTCCC**TCAGTTTTGCCCTATTTGCACA**AATT**TCAGTTTTGCCCTATTTGCACA**GGGTCCC**TCAGTTTTGCCCTATTTGCACA**AATT**TCAGTTTTGCCCTATTTGCACA**GGGTCCC**TCAGTTTTGCCCTATTTGCACA**AATT**TCAGTTTTGCCCTATTTGCACA**GGGTCCC**TCAGTTTTGCCCTATTTGCACA**AATT**TCAGTTTTGCCCTATTTGCACA**GGGTCCC**TCAGTTTTGCCCTATTTGCACA**AATT**TCAGTTTTGCCCTATTTGCACA**GGGTCCC**TCAGTTTTGCCCTATTTGCACA**AATT**TCAGTTTTGCCCTATTTGCACA**GGGTCCC**TCAGTTTTGCCCTATTTGCACA**AATT**TCAGTTTTGCCCTATTTGCACA**GGGTCCC**TCAGTTTTGCCCTATTTGCACA**AATT**TCAGTTTTGCCCTATTTGCACA**GGGTCCC**TCAGTTTTGCCCTATTTGCACA**AATT**TCAGTTTTGCCCTATTTGCACA**GGGTCCC**TCAGTTTTGCCCTATTTGCACA**AATT**TCAGTTTTGCCCTATTTGCACA**GGGTCCCGACGTTTAAACGAC*GAATTC* |
| miR-17-B sponge  (12xMBS) | *CTCGAG*GACG**CTACCTGCACTCCGGCACTTTG**GGAA**CTACCTGCACTCCGGCACTTTG**GACG**CTACCTGCACTCCGGCACTTTG**GGAA**CTACCTGCACTCCGGCACTTTG**GACG**CTACCTGCACTCCGGCACTTTG**GGAA**CTACCTGCACTCCGGCACTTTG**GACG**CTACCTGCACTCCGGCACTTTG**GGAA**CTACCTGCACTCCGGCACTTTG**GACG**CTACCTGCACTCCGGCACTTTG**GGAA**CTACCTGCACTCCGGCACTTTG**GACG**CTACCTGCACTCCGGCACTTTG**GGAA**CTACCTGCACTCCGGCACTTTG**GACGGAGCTCGGGCCC*GAATTC* |
| miR-18a-B sponge  (10xMBS)* | *CTCGAG*CCGG**TATCTGCACTTAGGCACCTTA**CCGG**TATCTGCACTTAGGCACCTTA**CCGG**TATCTGCACTTAGGCACCTTA**CCGG**TATCTGCACTTAGGCACCTTA**CCGG**TATCTGCACTTAGGCACCTTA**CCGG**TATCTGCACTTAGGCACCTTA**CCGG**TATCTGCACTTAGGCACCTTA**CCGG**TATCTGCACTTAGGCACCTTA**CCGG**TATCTGCACTTAGGCACCTTA**CCGG**TATCTGCACTTAGGCACCTTA**CCGGATCGCGGGCCC*GTTTAAAC* |
| miR-20a-B sponge  (7xMBS)* | *CTCGAG*CCGG**TACCTGCACTCGCGCACTTTA**CCGG**TACCTGCACTCGCGCACTTTA**CCGG**TACCTGCACTCGCGCACTTTA**CCGG**TACCTGCACTCGCGCACTTTA**CCGG**TACCTGCACTCGCGCACTTTA**CCGG**TACCTGCACTCGCGCACTTTA**CCGG**TACCTGCACTCGCGCACTTTA**CCGGATCGCGGGCCC*GTTTAAAC* |
| miR-92-B sponge  (10xMBS) | *CTCGAG*CTGGTTAACGACGGGTC**ACAGGCCGGTTTAGTGCAATA**GGTC**ACAGGCCGGTTTAGTGCAATA**GGTC**ACAGGCCGGTTTAGTGCAATA**GGTC**ACAGGCCGGTTTAGTGCAATA**GGTC**ACAGGCCGGTTTAGTGCAATA**GGTC**ACAGGCCGGTTTAGTGCAATA**GGTC**ACAGGCCGGTTTAGTGCAATA**GGTC**ACAGGCCGGTTTAGTGCAATA**GGTC**ACAGGCCGGTTTAGTGCAATA**GGTC**ACAGGCCGGTTTAGTGCAATA**GGTCCCGACGTTTAAACGAC*GAATTC* |
| miR-155-B sponge  (14xMBS) | *CTCGAG*CTGGTTAACGACGGGTCC**ACCCCTATGGAATTAGCATTAA**AATT**ACCCCTATGGAATTAGCATTAA**GTCC**ACCCCTATGGAATTAGCATTAA**AATT**ACCCCTATGGAATTAGCATTAA**GTCC**ACCCCTATGGAATTAGCATTAA**AATT**ACCCCTATGGAATTAGCATTAA**GTCC**ACCCCTATGGAATTAGCATTAA**AATT**ACCCCTATGGAATTAGCATTAA**GTCC**ACCCCTATGGAATTAGCATTAA**AATT**ACCCCTATGGAATTAGCATTAA**GTCC**ACCCCTATGGAATTAGCATTAA**AATT**ACCCCTATGGAATTAGCATTAA**GTCC**ACCCCTATGGAATTAGCATTAA**AATT**ACCCCTATGGAATTAGCATTAA**GTCCCGACGTTTAAACGAC*GAATTC* |
| Combi-sp1  (4x3xMBS) | *CTCGAG*TTAT**CTACCTGCACTCCGGCACTTTG**TTAT**CTACCTGCACTCCGGCACTTTG**TTAT**CTACCTGCACTCCGGCACTTTG**TTAT**CTATCTGCACTCAGGCACCTTA**TTAT**CTATCTGCACTCAGGCACCTTA**TTAT**CTATCTGCACTCAGGCACCTTA**TTAT**TCAGTTTTGCAGAGTTTGCACA**TTAT**TCAGTTTTGCAGAGTTTGCACA**TTAT**TCAGTTTTGCAGAGTTTGCACA**TTAT**ACAGGCCGGGTTCGTGCAATA**TTAT**ACAGGCCGGGTTCGTGCAATA**TTAT**ACAGGCCGGGTTCGTGCAATA**TTAT*GAATTC* |
| Combi-sp2  (4x3xMBS) | *CTCGAG*GCGG**CTACCTGCACTCCGGCACTTTG**GCGG**CTACCTGCACTCCGGCACTTTG**GCGG**CTACCTGCACTCCGGCACTTTG**GCGG**CTATCTGCACTCAGGCACCTTA**GCGG**CTATCTGCACTCAGGCACCTTA**GCGG**CTATCTGCACTCAGGCACCTTA**CGCC**TCAGTTTTGCAGAGTTTGCACA**CGCC**TCAGTTTTGCAGAGTTTGCACA**CGCC**TCAGTTTTGCAGAGTTTGCACA**CGCC**ACAGGCCGGGTTCGTGCAATA**CGCC**ACAGGCCGGGTTCGTGCAATA**CGCC**ACAGGCCGGGTTCGTGCAATA**CGCC*GAATTC* |
| Combi-scr  (4x3xMBS) | *CTCGAG*TTAT**CTACCTGCACTCCGATGCTCTG**TTAT**CTACCTGCACTCCGATGCTCTG**TTAT**CTACCTGCACTCCGATGCTCTG**TTAT**CTATCTGCACTCAGACGCTCTA**TTAT**CTATCTGCACTCAGACGCTCTA**TTAT**CTATCTGCACTCAGACGCTCTA**TTAT**TCAGTTTTGCAGAGTACGGTCA**TTAT**TCAGTTTTGCAGAGTACGGTCA**TTAT**TCAGTTTTGCAGAGTACGGTCA**TTAT**ACAGGCCGGGTTCATGACGTA**TTAT**ACAGGCCGGGTTCATGACGTA**TTAT**ACAGGCCGGGTTCATGACGTA**TTAT*GAATTC* |

XhoI (CTCGAG), EcoRI (GAATTC) or PmeI (GTTTAAAC) sites are in italic, each sponge consists of repeats of miRNA binding sites in bold followed by stuffer sequences in normal font. MBS = miRNA binding site. * miR-18a and miR-20a sponges were previously published by Ebert et al. (Nature Methods, 2007).
